# Advances in the Management of Fluid Resuscitation in Acute Pancreatitis: A Systematic Review

**DOI:** 10.3390/diagnostics15070810

**Published:** 2025-03-22

**Authors:** Cristian-Nicolae Costea, Cristina Pojoga, Andrada Seicean

**Affiliations:** 1 Departament of Gastroneterology, Iuliu Hațieganu University of Medicine and Pharmacy, Croitorilor Str., no 19-21, 400162 Cluj-Napoca, Romania; drcosteacristian@gmail.com; 2 Regional Institute of Gastroenterology and Hepatology, Croitorilor Str., no 19-21, 400162 Cluj-Napoca, Romania; cristinapojoga@yahoo.com

**Keywords:** acute pancreatitis, fluid resuscitation, systematic review

## Abstract

**Background/Objectives:** Acute pancreatitis (AP) is an inflammatory condition with diverse origins, often resulting in significant morbidity and mortality due to systemic inflammatory response syndrome (SIRS) and multiorgan failure. Fluid resuscitation is pivotal in early management, and it is aimed at preventing hypovolemia-induced ischemia and necrosis. This review evaluates fluid therapy strategies in AP, including fluid types, resuscitation rates, and clinical outcomes. **Methods:** This systematic review was conducted in January 2025 using databases such as PubMed, Medline, and Google Scholar, focusing on studies published between 2010 and 2024. Search terms included “acute pancreatitis”, “fluid resuscitation”, and related keywords. Studies involving adults with AP were analyzed to compare the outcomes of crystalloid and colloid use, aggressive vs. moderate fluid resuscitation, and administration timings. The primary outcomes were mortality and severe complications, while secondary outcomes included organ failure, SIRS, and length of hospital stay. **Results:** Crystalloids, particularly Ringer’s lactate (RL), are superior to normal saline in reducing SIRS, organ failure, and intensive care unit stays without significantly affecting mortality rates. Colloids were associated with adverse events such as renal impairment and coagulopathy, limiting their use. Aggressive fluid resuscitation increased the risk of fluid overload, respiratory failure, and acute kidney injury, particularly in severe AP, while moderate hydration protocols achieved comparable clinical outcomes with fewer complications. **Conclusions:** Moderate fluid resuscitation using RL is recommended for managing AP, balancing efficacy with safety. Further research is needed to establish optimal endpoints and protocols for fluid therapy, ensuring improved patient outcomes while minimizing complications.

## 1. Introduction

Acute pancreatitis is a rapidly evolving inflammatory disorder with various origins, including alcohol intake, biliary obstruction, autoimmunity, drugs, trauma, iatrogenic factors, and idiopathic conditions [1,2]. This inflammatory condition often culminates in organ failure, which is attributed to a systemic inflammatory response cascade, and it is associated with mortality rates of up to 40% [1,2,3,4].

The pathophysiology of AP involves the release of pro-inflammatory mediators, encompassing zymogens, cytokines, chemokines, and vasoactive factors [1,3,5]. Consequently, endothelial activation and arteriolar vasoconstriction occur, resulting in heightened permeability, circulatory stasis, and ischemia [1,2,3]. Intravascular fluid loss from capillary leakage contributes to hypotension, a reduction in the microcirculation of the pancreas, which could be an essential factor in the development of necrotizing pancreatitis. Hypovolemia results in splanchnic vasoconstriction and can provoke distributive shock, worsening tissue hypoxia and fostering systemic inflammatory response syndrome (SIRS) and multiple organ failure (MOF) [1,3]. Early management includes fluid resuscitation, pain management, and early oral feeding. Adequate pain management, typically with opioid or non-opioid analgesics, is essential to improve patient comfort without delaying enteral nutrition. Moreover, early oral feeding is now recommended over prolonged fasting, as it helps maintain gut integrity, reduces the risk of infections, and is associated with improved clinical outcomes [6,7].

Early fluid replacement has one of the most crucial roles in determining the evolution of AP because hypovolemia secondary to fluid loss increases the reperfusion of the pancreas, which results in ischemia and hypercoagulability statuses, followed by the local and systemic delivery of pro-inflammatory cytokines, resulting in severe pancreatitis [8,9].

The predictive markers for AP severity are as follows: age > 55 years old, obesity, SIRS, thoracic or abdominal pathological findings, and hematocrit of > 44%. Moreover, if hematocrit levels fail to decrease within the first 24–48 h, this is associated with evolution to necrosis and MOF [3,7], although controversial data have been published [10]. Other factors are serum urea levels of > 20 mg/dL and creatinine levels of > 2 mg/dL [7,11,12], while an elevated serum creatinine to bilirubin ratio is associated with more severe disease progression [13].

Fluid resuscitation should be chosen with regard to the following factors: fluid type, when to administer it, how long it takes to administer it, and methods for monitoring the results. However, there are differences between different guidelines (Table 1). All guidelines recommend using crystalloids, and the European guidelines are more specific about using RL.

There are two types of solutions used for fluid resuscitation: colloids with very high molecular weights—such as semisynthetic [hydroxyethyl starch (HES), gelatin, and dextran solutions] and natural (albumin) materials [3]—and crystalloids—such as normal saline (NS) and buffered solutions such as Ringer lactate (RL), Ringer’s ethyl pyruvate, Plasma-Lyte, or Hartmann’s solution.

High-rate fluid therapy has the potential to cause fluid overload, precipitating or exacerbating heart and respiratory failure [22], and pancreatic necrosis is already present by the time of diagnosis; thus, higher fluid volumes cannot prevent necrosis at this stage [23,24].

The most critical guidelines from gastroenterology and emergency medicine societies highlight the uncertainty of fluid management in patients with acute pancreatitis, noting the lack of resuscitation protocols and emphasizing the discrepancy between the ideal solution for resuscitation and the infusion rate [7,14,15,16,17,18,19,20,21]. This systematic review evaluates the benefits and risks of different fluid therapy protocols, providing an update on intravenous fluid treatment strategies and considering the types of fluids, crystalloids vs. colloids, combinations of crystalloids with colloids, optimal quantities, infusion rates, and routes of fluid administration in patients with acute pancreatitis [25].

## 2. Materials and Methods

This systematic review was conducted in accordance with the Preferred Reporting Items for Systematic Reviews and Meta-Analyses (PRISMA) 2020 guidelines [26]. This study was not prospectively registered in a systematic review database. All study identification, screening, eligibility assessment, and inclusion steps followed the PRISMA methodology in order to ensure transparency and reproducibility.

### 2.1. Information Sources and Search Criteria

In January 2025, a systematic literature search was conducted on electronic databases such as PubMed (Bethesda, MD, USA), Embase (Amsterdam, The Netherlands), Cochrane Library (London, UK), Web of Science (Philadelphia, PA, USA), and registries such as ClinicalTrials.gov (Bethesda, MD, USA) and Cochrane Central (London, UK).Following a pre-defined search strategy, all original studies investigating fluid management in acute pancreatitis were identified. The search covered studies published from 31 January 2010 to 31 January 2025 using MeSH (Medical Subject Headings) terms, which included “acute pancreatitis”. This term was combined with the Boolean operator “AND/OR”, along with studies identified through the keywords “fluid resuscitation”, “fluid therapy”, “hemoconcentration”, “colloid”, “crystalloid”, “Normal Saline”, “sodium chloride”, “Ringer’s lactate”, “albumin”, “dextran”, “Hartman solution”, “hydroxyethyl starch (HES)”, and “practice guidelines”. A manual search for further relevant articles was also performed using references to the included articles.

We used the Population, Intervention, Comparison, Outcome (PICO) methodology to define our research question. The target population for these studies was adult patients (≥18 years) with acute pancreatitis diagnosed using standard criteria based on the presence of two or three criteria: amylase or lipase levels that were more than three times the upper limit of standard, consistent imaging, and epigastric abdominal pain.

The outcomes were defined as the development of moderately severe and severe AP, as defined in the Revised Atlanta Classification [27]; the overall mortality; the occurrence of local and systemic complications; the intensive care unit (ICU) stay and length of hospitalization (LOS); the development of systemic inflammatory response syndrome; and the median CRP at 24 h. For the first aim of our review, we included studies with fluid resuscitation using LR solutions, while the comparison comprised hydration with NS. The second aim was to examine the resuscitation of aggressive/moderate fluid rates. The third was to establish the duration of the fluid resuscitation.

### 2.2. Data Collection

Two authors independently reviewed the abstracts of the selected studies to determine their eligibility. Full articles of potentially eligible abstracts were selected for further assessment. The included studies compared different types of fluids (colloids versus crystalloids and different types of colloids or crystalloids), infusion rate administration (high-rate versus moderate-rate fluid resuscitation), and routes of administration. No automation tools were used in the screening process. Through the extensive research of English articles from various sources, we obtained 2517 citations. After thoroughly scrutinizing each citation to eliminate duplicates and irrelevant entries, we selected studies that met our inclusion criteria—control trials and cohort studies. These studies compared the use of colloids or crystalloids in the volume resuscitation of acute pancreatitis, aggressive or moderate fluid resuscitation rates in acute pancreatitis, or the use of normal saline and Ringer’s lactate in acute pancreatitis.

We focused on full articles and recent technical guidelines from major gastroenterology societies while excluding studies with diseases other than AP; irrelevant citations based on titles and abstracts; case reports, reviews, editorials, and comments; studies in the pediatric population; and experimental studies on animal models, in addition to studies lacking precise comparative interventions. We identified duplicate reports from the same survey and only included the most recent publication or the one with the most extended follow-up period. We extracted the following data from each study using an organized data collection form: title of the study, publication year, name of the first author, year of the study, number of participants, demographic data of participants, type of fluid use, and outcome measurement (outcomes included mortality, the total number of serious adverse events observed, organ failure, sepsis, presence of SIRS at 24 h post-treatment, reduction level of C-reactive proteins at 24 h, ICU transfer, and length of hospital stay).

Heterogeneity was assessed qualitatively by comparing the study’s design, population characteristics, intervention protocols, and outcome measures. Differences were explored through subgroup analyses based on the study’s design and intervention type.

**Figure 1:**
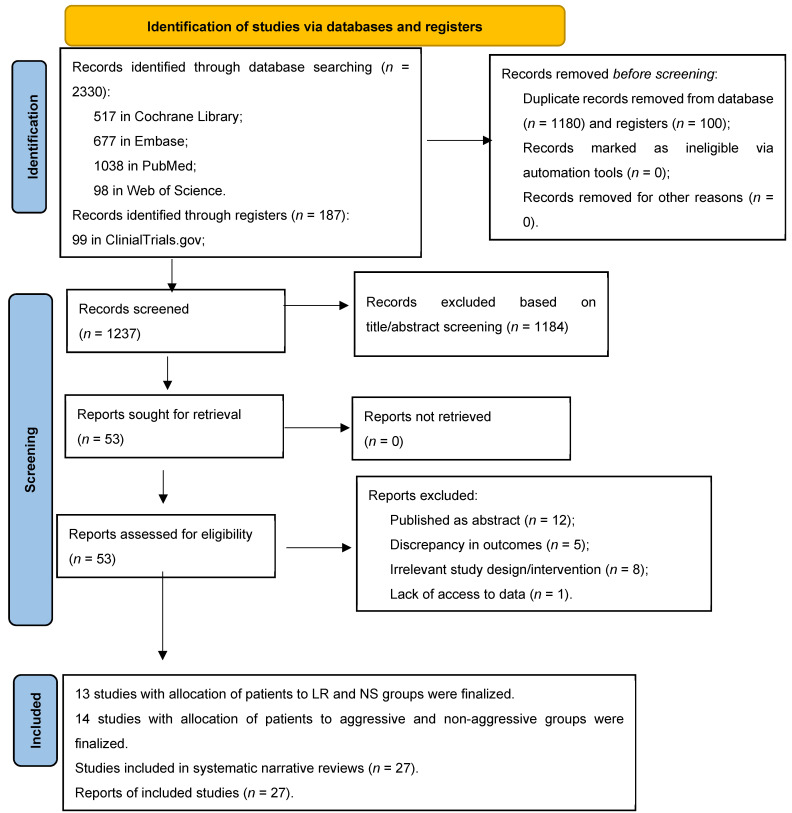
Flow chart of included studies. This flow diagram follows Prisma 2020 guidelines for systematic reviews [26].

The PRISMA flow diagram contains an overview of the detailed selection process [26,28] (Figure 1).

Two independent reviewers assessed the risk of bias using the Cochrane Risk of Bias 2.0 (RoB 2) tool. Discrepancies were resolved through discussion. A third reviewer adjudicated any disagreements if necessary. The risk of bias for randomized controlled trials was assessed using the Cochrane Risk of Bias 2.0 (RoB 2) tool, evaluating bias across five domains: randomization process, deviations from intended interventions, missing outcome data, measurement of outcomes, and selection of the reported results. Each study’s overall risk of bias was classified as low, with some concerns or high risk of bias. A sensitivity analysis was conducted to evaluate the potential impact of high-risk studies on the final conclusions.

## 3. Results

### 3.1. Timing for Starting Fluid Resuscitation

Fluid sequestration during the first 48 h after hospital admission varies between 1.4 L and 5 L, and it is higher in the presence of SIRS [29]. In a retrospective study of 340 patients with early fluid resuscitation and 94 patients with delayed resuscitation, the frequencies of SIRS and organ failure at 72 h were significantly lower (10% vs. 23% and 5% vs. 10%, respectively), with these phenomena occurring more frequently in patients with non-severe compared to severe pancreatitis at admission [30]. Another retrospective study proved that patients who do not receive at least one-third of their initial 72 h cumulative intravenous fluid volume during the first 24 h are at risk of more significant in-hospital mortality (18% vs. 0%) [31]. In summary, based on these outcomes, fluid resuscitation is likely more important early in the course of the disease—within the first 24 h—as mentioned in the current guidelines [7,20].

### 3.2. Fluid Type

- Ringer’s lactate (RL)

We found 13 studies consisting of 8 randomized control trials and 5 retrospective cohort studies (Table 2). RL prevents early organ failure by providing extracellular calcium and may have anti-inflammatory effects [32,33]. The main advantage of RL over NS is as follows: the decreased development of hyperchloremia [34] and the regulation of Toll-like receptor-mediated inflammation with a reduction in local inflammation around the pancreatic gland [35]. The results of an ongoing multicenter randomized controlled trial (Waterland trial) are anticipated to provide further evidence on the effectiveness of RL in acute pancreatitis, and its findings are eagerly awaited [36].

A meta-analysis of nine studies comprising 1424 AP patients in LR (*n* = 651) and NS (*n* = 773) with acute pancreatitis showed that patients were less likely to develop moderately severe/severe pancreatitis (OR = 0.48, 95%Cl 0.34 to 0.67; *p* < 0.001) and had fewer local complications (OR = 0.54, 95%Cl 0.32 to 0.92; *p* = 0.02) and shorter hospital stay lengths (−1.09 days; 95%Cl −1.72 to −0.47 days; *p* < 0.001) [37]. However, no differences in mortality rates between the RL and NS groups were observed [37]. A retrospective study involving 198 participants revealed a hospital mortality rate of 12% for all patients, with a lower rate of 5.8% in the LR group, while the NS group had a higher rate of 14.9% (OR = 3.10, *p* = 0.041) [38], which is different from other findings with respect to the death rate (25% in the RL group vs. 18% in the NS group) [26]. Other retrospective studies demonstrated lower mortality rates when LR was used compared to NS with respect to 1-year mortality (OR −0.61 [95%CI, 0.50–0.76]) [39] or in-hospital mortality (5.8% vs. 12.8%) in selected patients admitted to intensive care units [40].

The subgroup analyses of studies with SIRS at presentation were available only for six studies (Table 2), revealing a reduction in SIRS at 24 h when LR was used compared to patients treated with NS [12,34,41,42,43,44], but this was not confirmed in all studies [41,45] nor in a meta-analysis [24]. However, this beneficial effect was not mirrored at 48 h, showing no impact on disease-related mortality [46].

It was noted that the LR group had a shorter extended stay length in the ICU than the NS group [12,41,44]. Although there was a slight tendency toward shorter hospital stays for patients who received LR [12,34,38,43] or equal days of hospitalization [42,47], the difference was significant in a recent meta-analysis [35].

The inflammatory responses in AP lead to organ failure, and adverse outcomes [44,48] can be highlighted relative to the CRP level, which is associated with severity in AP [49]. The evolution of CRP levels at 24–72 h under fluid resuscitation was reported in only five studies [12,41,42,43,47], which proved that the administration of the LR solution also reduced the levels of CRP compared to NS at 48 h; however, a significant decrease was observed in only one study, without influence on clinical outcomes [43].

The risk of bias assessment indicated that most studies had a low to moderate risk of bias, particularly in the measurement and reporting domains. However, two studies [38,39] (Antoniak et al., 2024; Aboselsoud et al., 2016) exhibited a high risk of bias, and this was primarily due to inadequate randomization and missing outcome data, requiring the cautious interpretation of their findings.

**Table 2:** Main studies investigating fluid administration in acute pancreatitis.

Author, Year	Type of Study	No. of pts (n=)		Organ Failure %		Local Complications %		Mortality %		ICU Transfer %		LOS (Median, days)		Level of CRP at 24 h (mg/L)		SIRS Reduction at 24 h (%)	
		LR/ NS	P	LR/ NS	P	LR/NS	P	LR/ NS	P	LR/ NS	P	LR/NS	P	LR/ NS	P	LR/ NS	P
Wu et al., 2011 [12]	RCT	19/ 21	Ns	5/ 20	ns	0/ 15	ns	0/ 0	ns	5/ 14	ns	5/ 5.5	ns	51.5/ 104	0.02	84/ 0	0.035
Lipinski et al., 2015 [50]	R	40/ 63	ns	N/A	N/A	10/ 12	ns	12.5/ 4.76	ns	N/A	N/A	9/ 9	0.776	N/A	N/A	N/A	N/A
Aboselsoud et al., 2016 [38]	R	68/ 130	ns	N/A	N/A	N/A	N/A	5.8/ 16.1	0.029	N/A	N/A	# 6.2/4.2	0.020	N/A	N/A	N/A	N
Mok SRS et al., 2017 [51]	RCT	48/ 48	ns	2/ 2	ns	0/ 0	ns	2 4	ns	N/A	N/A	4.3 /2.2	ns	N/A	N/A	N/A	N/A
De Madaria et al., 2018 [41]	RCT	19/ 21	ns	0/ 4.8	ns	40/ 71.4	ns	0/ 4.8	ns	0/ 4.8	ns	9/ 9	ns	* 28/ 166	0.037	21.1/ 19	ns
Choosakul et al., 2018 [42]	RCT	23/ 24	ns	12.6/4.3	ns	26.8/37.5	ns	0/ 4.2	ns	N/A	N/A	6/ 5.5	ns	* 18.9/31.7	ns	8.7/ 37.5	0.02
Lee et al., 2021 [34]	RCT	61/ 60	ns	11.5/ 15	ns	6.6/15	ns	0/0	ns	9.8/ 25	ns	3.5/4.6	ns	N/A	N/A	37.5/ 32.2	ns
Kayhan et al., 2021 [47]	RCT	67/ 65	ns	31.6/68.4	ns	38.9/61.1	ns	N/A	N/A	N/A	N/A	3/ 3	ns	* 26.0/73.7	0.010	N/A	N/A
Karki et al., 2022 [43]	RCT	26/ 25	ns	N/A	N/A	23.1/28	ns	0/ 4	ns	N/A	N/A	5.15/6.2	ns	** 14.2/ 22.2	<0.001	15.4/ 44.0	0.025
Lee PJ et al., 2023 [44]	R	328/ 364	ns	5.2/ 3.9	ns	N/A	N/A	N/A	N/A	7.3/ 7.1	ns	N/A	N/A	N/A	N/A	56.2/ 0	0.011
Horibe M et al., 2024 [40]	R	8053/657	ns	50/66.3.7	ns	4.2/4.8	ns	8.5/ 12.8	ns	4/5	ns	20/ 23	ns	N/A	N/A	28.7/ 40.1	0.008
Farrell PR et al., 2024 [46]	RCT	38/ 38	ns	N/A	N/A	8/ 8	ns	N/A	N/A	N/A	N/A	67.5/80	ns	* 2.9/1.8	ns	* 16/ 6	ns
Antoniak et al. 2024 [39]	R	2049/ 18,022	ns	N/A	N/A	21.4/ 21.1	ns	2/ 1.7	ns	0.8/ 0.6	ns	3.7/ 3.7	ns	N/A	N/A	19/ 21.1	0.03

LR—lactated Ringers; NS—normal saline; ns—non-significant; N/A—not available; ICU—intensive care unit; *—at 48 h; **—at 72 h; LOS—hospital length of stay; CRP—C-reactive protein; #—Intensive Care Unit LOS.

- HES (hydroxyethyl starch)

Few data studies consider the use of HES. A randomized controlled trial compared HES resuscitation with an RL group, and they reported a slight decrease in intra-abdominal pressure and a need for mechanical ventilation in the HES group, with a realization of negative fluid balance [52].

Another retrospective study on 120 severe AP patients showed that renal functions and immune statuses were preserved after HES therapy [53]. However, a meta-analysis proved that when resuscitation with NS versus HES was compared, organ failure occurrence was higher in patients treated with HES (RR 0.30; 95%IC 0.21–0.44, *p* < 0.001)), while NS and RL showed reduced SAEs in comparison with HES (RR 0.38; 95%IC 0.27–0.54, *p* < 0.001 and RR = 0.53, 95%IC 0.19–1.43, respectively) [54]. Moreover, the mortality rate was higher in the HES groups compared to the NS group (RR = 2.71, 95%IC 0.49–14.9) [55]. Therefore, based on these findings, HES administration cannot be recommended with respect to the current guidelines [7,14,20].

- Albumin

A retrospective study proved that administrating exogenous albumin to maintain serum levels at 3.7–4 g/dL during the first 28 days after the onset of AP alleviates prognosis [52], but this was not sustained in other prospective studies [55]. Albumin diluted with dextran reduced mortality rates by 7.7% and slowed the progression of pancreatic necrosis by 15.0% [56].

- Glucose-based fluid resuscitation

A multicenter retrospective cohort study investigated the impact of glucose-containing fluids on AP outcomes. Among 1146 patients, excess glucose-containing fluids were associated with higher risks of overall, respiratory, and cardiovascular organ failure, in addition to increased ICU admissions [45], suggesting that these fluids cannot be chosen for fluid resuscitation.

- Combination of Ringer lactate solution and human albumin

A randomized clinical trial involving 300 participants evaluated whether aggressive intravenous hydration with lactated Ringer’s solution and 20% human albumin (intervention group) prevents post-ERCP pancreatitis compared to those who received standard-volume intravenous hydration with lactated Ringer’s solution (control group) prior to ERCP. The study found that aggressive intravenous hydration with lactated Ringer’s solution and 20% human albumin did not significantly reduce post-ERCP pancreatitis (6.7% vs. 6.3%, *p* = 0.873) [57].

In summary, current evidence supports the use of LR over NS due to its anti-inflammatory properties and potential benefits in lowering SIRS and shortening ICU stays. However, mortality benefits remain inconsistent, and further research, such as the ongoing Waterland trial, is awaited in order to confirm its role in acute pancreatitis management. Hydroxyethyl starch (HES), albumin, and glucose-based fluids remain controversial, with limited supporting evidence and potential risks.

### 3.3. Fluid Resuscitation Endpoint

The vigilant monitoring of rehydration and cardiovascular, renal, and respiratory functions is essential for the early detection of electrolyte imbalances and volume overload, especially in elderly patients and those with cardiovascular and renal comorbidities [58]. Monitoring central venous pressure is generally not needed [59,60].

- After 24 h from admission, the suggested goals are as follows:
- Heart rate (HR) < 120 bpm;
- Mean arterial pressure (MAP) > 65 mmHg;
- Urinary output (UO) > 0.5 mL/kg/h;
- Ht 35–44%;
- Normal blood urea nitrogen concentration [21,26,48,60,61,62,63,64,65,66,67].

Given that most patients with AP typically exhibit mild disease, clinicians may underestimate the necessity of early hydration, particularly since these patients often do not appear very ill, exhibiting normal hematocrit (HCT) and blood urea nitrogen (BUN) levels. It may seem that the treatment goals for these patients are being achieved; however, AP can cause significant intravascular fluid to leak into the peritoneum, averaging 2–4 L during the first 48 h. If moderately aggressive intravenous hydration is not initiated in patients who appear stable, especially if their hematocrit increases within the first 24–36 h, the treatment objective can be missed, resulting in heightened risks of necrosis and/or organ failure. Rather than viewing intravenous hydration as goal-directed therapy, it is more appropriate to consider it essential therapy, and the following principle should be followed: do not let urea and hematocrit levels rise in the first 24–48 h or allow the development of SIRS and/or renal insufficiency [67].

Moreover, aggressive fluid resuscitation produces adverse effects on the respiratory and renal system [68] or could result in compartment syndrome, and it is associated with an increased risk of death in critical care settings [18,58,69]. In the recently published WATERFALL study (halted early due to safety concerns), higher fluid overloads occurred in 20.5% of patients with aggressive fluid resuscitation compared to 6.3% in the other group [22]; thus, clinicians should monitor edema, swelling, and local infection signs [22].

In summary, optimal fluid resuscitation should be goal-directed—monitoring the goals already mentioned—to prevent under-resuscitation or over-resuscitation. Clinicians should be cautious in patients with cardiovascular or renal comorbidities in order to avoid complications associated with fluid overload.

### 3.4. Fluid Rate

There are still many controversies regarding the ideal rhythm of fluid resuscitation in AP. The current recommendations are as follows: start with a bolus of 10 mL fluid/kg, followed by moderately aggressive fluid resuscitation with 1.5 mL/kg/h [23]. Early moderate aggressive fluid therapy among patients with predicted mild severity (10 mL/kg followed by 1.5 mL/kg/h to 5–10 mL/kg/h) appears to be the most beneficial. In contrast, aggressive resuscitation (20 mL/kg bolus followed by 3 mL/kg/h) in patients with predicted severe disease might be futile and deleterious. Moreover, the clinical evolution of AP patients at more than 24 h from disease onset is similar using aggressive or non-aggressive hydration [59].

We found fourteen studies, which comprised seven randomized control trials and one prospective and six retrospective cohort studies, and they were obtained from various databases mentioned in the reference (Table 3). A meta-analysis including six RCTs proved that mortality is higher in aggressive fluid therapy (RR 2.40, CI: 1.38–4.19) no matter the patient’s age or initial AP severity, with no differences in other clinical outcomes—such as the length of hospital stay and the development of SIRS or organ failure—compared to a strategy of moderate fluid replacement [24]. In the Asian population, the mortality risk for aggressive fluid therapy was significant only in severe AP [70], but further assessments are needed from other populations to clearly establish the role of AP severity. An artificial intelligence system (ADAPT system), in which data collected retrospectively were introduced (pancreatitis etiology, gender, and SIRS at admission), was used to generate resuscitation fluid recommendations, which were compared to actual fluid resuscitation practices. Of 1083 patients, almost 70% were over-resuscitated, and this was correlated with increased respiratory failure (OR = 2.73 [95%CI 1.06, 7.03]), higher ICU admission (OR 2.40 [1.41, 4.11]), longer hospital length of stay (OR 1.87 [1.19, 2.94]), and higher local pancreatitis complications (OR 2.93 [1.23, 6.96]) [71]. Separating studies comparing high (≥20 mL/kg/h), moderate (≥10 to <20 mL/kg/h), and low (5 to <10 mL/kg/h) fluid therapy, low-volume fluid resuscitation was associated with better clinical outcomes but higher mortality, systemic complications, and SIRS persistence than moderate or high fluid therapy [72].

An increased risk of sepsis in the aggressive intravenous hydration group was found in severe AP [70,73]. Moreover, rapid hemodilution could increase the incidence of sepsis and in-hospital mortality within 28 days, and hematocrit should be maintained between 30% and 40% in the acute response stage [73]. An increased rate of noninvasive positive-pressure ventilation was reported in a prospective trial that included hemoconcentration patients with severe AP, but no clinical impacts were observed with respect to hemoconcentration, non-severe AP, or non-hemoconcentration AP patients [74].

Li Lan et al. (2020) [74] and Mao et al. (2010) [73] were identified as high-risk studies due to unclear allocation concealment and missing data issues in the analysis of fluid resuscitation strategies. Despite these concerns, the overall conclusions remained stable, as confirmed by a sensitivity analysis.

In summary, moderate fluid resuscitation (1.5 mL/kg/h to 5–10 mL/kg/h) is generally recommended, while aggressive fluid therapy (>3 mL/kg/h) may increase mortality and complications without clear benefits. Over-resuscitation is related to respiratory failure, sepsis, and ICU admission. A balance between adequate volume replacement and the avoidance of fluid overload should be sought, particularly in severe cases.

**Table 3:** Summary of studies comparing different intravenous fluid resuscitation strategies with respect to acute pancreatitis (author, year).

Author, Year	Type of Study	No. of pts	Aggressive vs. Non-Aggressive		Resuscitation Endpoint	Study Endpoint	Results
			Rate	Amount			
Tomanguillo J. et al., 2023 [61]	R	10,400	>3 mL/kg/h vs. ≤1.5 mL/kg/h	N/A	- Mortality	- 30-day mortality - MV rates - Severe sepsis	No Δ in mortality and LOS.
de-Madaria et al., 2022 [22]	RCT	249	20 mL/kg bolus then 3 mL/kg/h vs. 10 mL/kg bolus then 1.5 mL/kg/h	5400 mL vs. 3310 ml	UO, signs of dehydration, SBP, fluid overload	- Development of moderate–severe/severe AP during hospitalization - Fluid overload	No Δ outcome Higher incidence of fluid overload in the aggressive resuscitation group.
Angsubhakorn et al., 2021 [62]	RCT	44	20 mL/kg bolus then 3 mL/kg/h vs. 10 mL/kg bolus then 1.5 mL/kg/h	4886 mL vs. 3985 ml	BUN, Ht, UO, Cr oral tolerance, and clinical examination	- Clinical improvement within 36 h - Decrease in Ht, BUN, and Cr and reduced epigastric pain level tolerance of oral intake, development of SIRS	No Δ clinical outcomes in mild AP but improvement only in patients with obese status, not sustained at 36 h.
Sperna Weiland CJ., 2022 [75]	RCT	826	20 mL/kg, then 3 mL/kg/h for 8 h. vs. 1·5 mL/kg/h and 3 L/24 h.	N/A	N/A	Post-ERCP AP	Aggressive periprocedural hydration to further reduce the risk of post-ERCP AP is not justified.
Messallam Ahmed A et al., 2021 [76]	R	310	4.475 L/24 h vs. <2.8 L/24 h	N/A	- Total IV fluids in the first 24 h	- OF - In-hospital mortality	Aggressive FT increased persistent OF and LOS without lowering mortality.
Cuéllar-Monterrubio JE et al., 2020 [59]	RCT	88	20 mL/kg bolus then 3 mL/kg/h for the first 24 h, and then 30 mL/kg for 24 h vs. 1.5 mL/kg/h for the first 24 h and 30 mL/kg during 24 h	6400 mL vs. 2795 ml	Vital signs, UO, BUN, Ht, lactate, SIRS	- Development of SIRS, OF - LOS	No Δ in outcome.
Li Lan et al., 2020 [74]	R	912	≥3 mL/kg/h vs. <3 mL/kg/h	N/A	Ht > 44%	Rate of MV, LOS	MV and longer LOS associated with rapid FT in severe AP and Ht ≥ 44%.
Ye Bo et al., 2018 [60]	R	179	N/A	>4 L/day vs. <4 L/day	Vital signs, UO, Ht	AKI development/chloride exposure	Aggressive FT increased the risk and duration of AKI, a high-chloride exposure risk factor for new-onset AKI.
Yamashita T et al., 2019 [70]	R	1097	N/A	≥ 6 L/day vs. <6 L/day	N/A	In-hospital mortality	Higher mortality in unadjusted analysis but lower mortality in adjusted analysis.
Park CH et al., 2018 [77]	RCT	395	3 mL/kg/h during ERCP, 20 mL/kg bolus after ERCP, and then 3 mL/kg/h for 8 h vs. 1.5 mL/kg/h during and for 8 h after ERCP	N/A	Post-ERCP AP	- Incidence of post-ERCP AP - Post-ERCP hyperamylasemia, epigastric pain, and fluid overload	Aggressive hydration with LRS is the best approach to intravenous hydration to prevent post-ERCP AP in average-to-high-risk patients.
Singh et al., 2017 [64]	R	1010	>1 l in first 4 h or >4.3 L in first 24 h vs. <0.5 L in first 4 h or <3.2 L in first 24 h	>4300 mL; >1000 mL in the first 4 h vs. <3200 mL, <500 mL for the first 4 h	N/A	- Need for invasive interventions - Local complications - Persistent OF - Mortality	No Δ impact mortality or OF. Early moderate to aggressive FT in the emergency room, within 4 h from admission, was associated with a lower need for invasive interventions.
Buxbaum et al., 2017 [65]	RCT	60	20 mL/kg bolus then 3 mL/kg/h vs. 10 mL/kg bolus then 1.5 mL/kg/h	5600 mL vs. 3900 mL	Ht, BUN, Cr, pain score	- Decrease in Ht, BUN, Cr - Reduced pain - Tolerance to oral feeding - Development SIRS	Higher clinical improvements; reducing SIRS and hemoconcentrations in the aggressive group in mild AP may not apply to severe AP.
de-Madaria et al., 2011 [78]	P	247	N/A	Initial 24 h: <3.1 L vs. initial 24 h: >4.1 L	Ht, UO, SBP, Cr, SIRS	- Persistent OF lasting > 48 h - Local complications - Mortality	>4.1 L was linked to worse outcomes, including increased OF and local complications. <3.1 L was not associated with OF, local complications, or mortality. 3.1 L–4.1 L had an excellent outcome.
Mao et al., 2010 [73]	RCT	115	10–15 mL/kg/h vs. 5–10 mL/kg/h	6855 mL vs. 5841 mL	Ht<>35%, Cr, BUN	- In-hospital mortality - Incidence of sepsis - OF	Rapid hemodilution can increase the incidence of sepsis within 28 days and in-hospital mortality.

N/A—not available; AP—acute pancreatitis; BUN—blood urea nitrogen; Ht—hematocrit; Cr—serum creatinine; AKI—acute kidney injury; FT—fluid therapy; LOS—hospital length of stay; SIRS—systemic inflammatory response syndrome; Δ—indicates change; UO—urine output; SBP—systolic blood pressure; OF—organ failure; MV—mechanical ventilation; R—retrospective cohort; P—prospective trial; RCT—randomized clinical trial.

## 4. Discussions

Fluid resuscitation remains a cornerstone in the early management of acute pancreatitis (AP), with fluid therapy’s timing, type, rate, and endpoints crucial to patient outcomes.

According to the latest ACG guideline [7], treating a patient with mild AP is beneficial because endothelial injuries and increased vascular permeability are present, which can result in moderate to severe forms of AP. Intravenous hydration aims to impede the increased AP severity because it can reverse the decrease in pancreatic blood flow and prevent pancreatic cellular death, necrosis, the ongoing release of pancreatic enzymes, and the inflammations responsible for fluid loss in the third space [3].

Early fluid resuscitation, initiated within the first 24 h, is crucial in preventing disease progression in acute pancreatitis [7]. Delayed resuscitation has been associated with increased SIRS, organ failure, and higher mortality rates [31,32]. Starting early after admission within the first 24 h, individualized hydration strategies should be guided by patient response and disease severity, with the aim of reducing the risk of SIRS, organ failure, and mortality.

The fluid types used for resuscitation introduce considerable debates among specialists.

NS has some limitations because it produces high chlorine concentrations with metabolic acidosis that could lead to increased inflammatory responses and, subsequently, an increase in pancreatitis severity [79]. Additionally, adverse effects related to hyperchloremia and metabolic acidosis could result in acute kidney injury due to a decrease in renal blood flow and renal vasoconstriction [56,80].

The advantage of RL is related to its pH balance because it has a lower chloride concentration than NS, and it contains bicarbonates and lactates, which may prevent metabolic acidosis, resulting in an anti-inflammatory effect and a better outcome for AP patients [54].

Colloids comprise large molecules suspended in a carrier solution, and they remain in the intravascular compartment longer than in crystalloid solutions, resulting in high oncotic pressure and maintaining the intravascular volume. Despite some improvements in pancreatic circulation and immunomodulation [81,82], colloids have increased adverse effects: volume overload, hyper-oncotic renal impairment, coagulopathy, anaphylactic reaction [8,83], or pulmonary edema [3,81]. Colloids are considered in anemia Ht < 25% and hypoalbuminemia < 2 g/dL [84].

Among fluid types, LR is preferred over NS due to its anti-inflammatory properties, ability to reduce hyperchloremia, and potential benefits in lowering ICU stays [37] and mortality, although future research results are needed [36].

The latest guideline does not recommend colloids as the initial fluid of choice for resuscitation in the acute inflammatory phase of AP [18], with a special mention of HES, which should be avoided due to renal impairment in patients with sepsis [22]. Although some studies have suggested a possible combination of crystalloids and colloids, research in this direction is scarce [8,25,85].

Moderate fluid resuscitation (1.5–5 mL/kg/h) is generally recommended, as aggressive hydration (>3 mL/kg/h) is associated with higher mortality, respiratory failure, and increased ICU admissions without significant clinical benefits [24]. Goal-directed resuscitation should be emphasized, with the careful monitoring of heart rates, mean arterial pressure, urine output, hematocrit levels, and blood urea nitrogen to balance adequate volume replacement and the risk of fluid overload [22].

Although this was not the aim of our review, fluid therapy plays a crucial role in the prevention of post-endoscopic retrograde cholangiopancreatography pancreatitis (PEP).

Preventing PEP is a critical consideration in clinical practice. Two primary prophylactic strategies—the rectal administration of non-steroidal anti-inflammatory drugs (NSAIDs), such as indomethacin, and prophylactic pancreatic duct (PD) stent placement—have been extensively studied [86,87] with respect to high-risk individuals, particularly those undergoing pancreatic duct cannulation or presenting multiple-risk factors [7,51,86,87,88]. Recent evidence suggests that combining rectal NSAIDs with prophylactic PD stenting may offer additional protective benefits against PEP [88,89]. A randomized controlled trial (RCT) demonstrated that LR is preferred over NS for aggressive hydration during ERCP for prophylactic purposes (10 mL/kg bolus followed by continuous infusion at 3 mL/kg/h), as it significantly reduces and enhances pancreatic microcirculation [90] and has potential anti-inflammatory effects [77].

This conclusion is further supported by a meta-analysis that included ten RCTs with a total of 2200 patients, demonstrating that aggressive hydration significantly reduced the incidence of PEP ([OR, 0.40; 95% CI, 0.26–0.63; *p* < 0.0001) compared to standard hydration. However, there was no significant difference in adverse events between aggressive and standard hydration [90].

Caloric requirements play a critical role in managing acute pancreatitis, as energy balance directly influences both fluid therapy needs and metabolic homeostasis, mainly because the patient cannot be fed orally within the first 24–48 h after admission. In critically ill patients, inadequate caloric intake can result in catabolism, muscle breakdown, and negative fluid balance, thereby increasing the need for aggressive fluid resuscitation to maintain hemodynamic stability [91]. Conversely, overfeeding should be avoided, which may exacerbate systemic inflammation [91]. Indirect calorimetry remains the gold standard for accurately measuring resting energy expenditure [92]; however, in its absence, predictive equations, such as the Harris–Benedict equation or those recommended by ESPEN, serve as practical alternatives [93,94]. Current guidelines suggest an energy target of 25–30 kcal/kg/day in critically ill patients, with a preference for enteral nutrition over parenteral nutrition to preserve gut integrity, reduce infectious complications, and improve clinical outcomes [94]. Enteral nutrition should be utilized from the start of hospitalization in all malnourished patients and patients with predictive factors for severe acute pancreatitis, and it should be utilized within 72 h in all patients in whom oral nutrition does not cover 60% of protein–calorie requirements [95]. 

Future studies should further explore the integration of individualized calorie assessments in early acute pancreatitis management.

This study has potential limitations, such as heterogeneity among the included studies, publication bias, or a lack of randomized controlled trials (RCTs) in certain areas.

## 5. Conclusions

Current recommendations for treating acute pancreatitis advocate administering a moderate volume of fluids intravenously. Aggressive fluid resuscitation is linked to a higher mortality risk in patients with severe acute pancreatitis and an increased chance of fluid-related complications in both severe and non-severe cases.

Crystalloids, particularly Ringer’s lactate (RL), are the preferred fluids. The recommended initial fluid administration rate usually ranges from 5 to 10 mL/kg/h during the first 24 h. Reducing the rate to 2–3 mL/kg/h is reasonable if resuscitation targets are met at any point within that period. Further research is also necessary to determine the volume of fluids needed and the duration of fluid resuscitation based on AP severity required in order to minimize the complications associated with under-resuscitation and over-resuscitation.

## Figures and Tables

**Table 1:** Comparison of fluid management guidelines in acute pancreatitis.

Guideline	Fluid Type Recommendation	Initial Resuscitation Rate	Resuscitation Goals
American College of Gastroenterology (ACG) (2024) [7]	Isotonic crystalloids (NS or LR)	250–500 mL/h	Improve hemodynamics, avoid over-resuscitation
British Society of Gastroenterology (BSG) (2023) [14]	LR (or Hartmann’s), goal-directed rehydration	5–10 mL/kg/h or until resuscitation goals reached	Prevent complications, optimize organ function
German Clinical Practice Guideline (2022) [15]	RL preferred	200–250 mL/h for 24 h	Reduce mortality, prevent hypoperfusion
Japanese Clinical Practice Guidelines (JPN) (2021) [16]	Goal-directed fluid therapy; no preference between LR and NS	Moderate resuscitation, avoiding overhydration	Maintain stable hemodynamics, prevent complications
World Society of Emergency Surgery (WSES) (2019) [17]	Balanced crystalloids preferred	No fixed rate; guided by dynamic assessment	Hemodynamic stabilization, lactate clearance
American Gastroenterological Association (AGA) (2018) [18]	Balanced crystalloids (LR preferred over NS)	5–10 mL/kg/h (moderate), avoiding aggressive states (>10 mL/kg/h)	Normalize hematocrit, BUN, urine output ≥ 0.5 mL/kg/h
European Society of Gastrointestinal Endoscopy (ESGE) (2018) [19]	RL	5–10 mL/kg/h	Tailored approach with frequent reassessment
Best Practice in General Surgery, Canada (2016) [20]	Balanced crystalloids, LR preferred	No specific rate mentioned; focus on clinical monitoring	Optimize fluid balance, prevent complications
International Association of Pancreatology (IAP)/American Pancreatic Association (APA) (2013) [21]	RL preferred	5–10 mL/kg/h	Reduce the risk of SIRS and organ failure

LR—lactated Ringers; NS—normal saline.

## Data Availability

No new data were created or analyzed in this study.

## Abbreviations

The following abbreviations are used in this manuscript:

N/A	Not available
AP	Acute pancreatitis
BUN	Blood urea nitrogen
LR	Lactated Ringers
NS	Normal saline
HES	Hydroxyethyl starch
ICU	Intensive care unit
CRP	C-reactive protein
FT	Fluid therapy
Ht	Hematocrit
Cr	Creatinine
AKI	Acute kidney injury
SIRS	Systemic inflammatory response syndrome
LOS	Hospital length of stay
OU	Urine output
Δ	Indicates change
ns	Non-significant
MOF	Multiple organ failure
SBP	Systolic blood pressure
OF	Organ failure
MV	Mechanical ventilation
R	Retrospective cohort
P	Prospective trial
PEP	Post-endoscopic retrograde cholangiopancreatography pancreatitis
PD	Pancreatic duct
RCT	Randomized clinical trial
